# Changes in Academic Performance after Transitioning to Remote Proctoring: A Before-After Evaluation

**DOI:** 10.3390/pharmacy10040092

**Published:** 2022-07-28

**Authors:** Elizabeth A. Hall, Madison B. Roberts, Katharyn A. Taylor, Dawn E. Havrda

**Affiliations:** Department of Clinical Pharmacy and Translational Science, College of Pharmacy, University of Tennessee Health Science Center, Memphis, TN 38163, USA; madison.roberts@vcuhealth.org (M.B.R.); ktaylo84@uthsc.edu (K.A.T.); dhavrda@uthsc.edu (D.E.H.)

**Keywords:** academic performance, testing anxiety, proctoring, assessment, COVID-19

## Abstract

Remote proctoring is often used to ensure testing integrity in a distance education environment but may impact academic performance. This quasi-experimental study aimed to evaluate changes in examination scores after transitioning to remote proctoring during the COVID-19 pandemic. Student pharmacists (*n* = 384) served as their own controls in this before-after analysis of examination scores with in-person versus remote proctoring. To assess differences in examination scores among students with varying levels of testing anxiety, students were classified into low, moderate, or high testing anxiety groups based on their Cognitive Test Anxiety Scale–Second Edition (CTAS-2) score. Students were also stratified into two groups based on their cumulative grade point average (GPA). After transitioning to remote proctoring, examination scores significantly decreased for first-year (P1) students but significantly increased for second-year (P2) students. When stratified by CTAS-2 score, no significant difference in examination scores was found. When stratified by GPA, no significant difference in examination scores was found for P1 students, but a significant improvement was noted for P2 students with remote proctoring. The results of this study indicate that examination scores do not consistently improve or decline after introducing remote proctoring even when considering a student’s GPA and level of testing anxiety.

## 1. Introduction

The COVID-19 pandemic has accelerated the pace of changes in education and learning and has forced educational institutions to rapidly transition out of the brick-and-mortar classroom and into a digital space [1,2,3]. While lectures are relatively simple to publish and access virtually, maintaining integrity for online student assessment and testing may be more challenging [4]. Research to date is sparse and inconclusive regarding whether academic dishonesty is more, less, or equally prevalent in an online environment [5,6,7]. However, online, high-stakes, summative examinations present two of three conditions that are positive predictive factors of student cheating behavior (i.e., opportunity via the examination’s online medium and pressure via the high-stakes nature of the assessment), thereby raising the concern for academic dishonesty [8].

Live remote proctoring during remotely administered examinations has been proposed to be the most rigorous method to ensure academic integrity [5]. As such, various software platforms exist to enhance at-home examination integrity. However, negative experiences, including lack of information technology (IT) support, lack of proper monitoring, and poor timeliness have been reported with these platforms [9]. Additionally, these platforms have a fee per use, which may further limit their utility. Given these potential limitations, a remote proctoring process was devised and implemented using only internal resources [10] rather than relying on an external platform after our institution transitioned to a distance learning model on 16 March 2020 due to the COVID-19 pandemic.

By the end of the spring 2020 semester, students were reporting increased stress and anxiety levels. Given the previously published findings of increased stress and anxiety leading to worse academic performance in student pharmacists [11,12] and medical sciences students [13], we hypothesized that the mid-semester transition to remote proctoring may have had a negative impact on a student’s academic performance.

This study aimed to evaluate the impact of a transition to remote proctoring on academic performance in first- (P1) and second-year (P2) Doctor of Pharmacy (PharmD) students. This study had two secondary aims: (1) to investigate whether academic performance after the transition to remote proctoring differed in cohorts of low, moderate, or high testing anxiety and (2) to examine whether effects on examination performance after transitioning to remote proctoring differed based upon the student’s overall academic performance, as indicated by cumulative grade point average (GPA).

## 2. Materials and Methods

### 2.1. Remote Proctoring Process

The remote proctoring process developed and implemented in this study only used internal resources and technology that were already available to the institution. Remote proctoring required student pharmacists to use two devices. On the first device, videoconferencing software (Zoom Video Communications, Inc., San Jose, CA, USA) was used to monitor the student’s exam-taking space. On the second device, also known as the student’s testing device, the computer-based exam was administered via ExamSoft (ExamSoft Worldwide, Inc., Dallas, TX, USA) in a secure manner. Secure exams in ExamSoft are set to restrict access to all other applications and disable all device functions during the examination.

Proctors consisted of UTHSC College of Pharmacy faculty and staff who had completed a one-hour orientation and training session to ensure standardization of procedures amongst all proctors. This training session outlined proper procedures before, during, and after the examination along with a written guide and checklist for additional help. In addition, one-on-one sessions with IT staff were available to proctors if needed for adequate orientation to using the software and related technologies. Student pharmacists also attended a one-hour informational session to learn what to expect on examination day. Like proctors, student pharmacists were also able to consult with IT staff as needed before the first remotely administered and proctored examination.

Prior to starting the examination, the proctor completed a check-in process with each student individually. This process included asking each student to show a 360° view of their workspace to ensure no unauthorized materials were present and an ID verification to ensure the correct identity of the exam taker. Throughout the session, students maintained audio and visual connections. Each proctor observed up to 24 students for the duration of the exam and noted potentially suspicious activities. Any suspicious activities were reported to the Office of Academic Affairs within the UTHSC College of Pharmacy. The UTHSC College of Pharmacy coordinator of testing was available to join the Zoom session and bring any student showing suspicious activities into a breakout room to actively intervene in real-time without interrupting other students. IT staff were available to help students and proctors with any technical difficulties during the examination.

### 2.2. Research Design and Setting

This quasi-experimental study evaluated composite examination scores before and after a transition to remote proctoring due to the COVID-19 pandemic. The study setting was a single 4-year, public PharmD program based in the Southeastern U.S. This study was reviewed by the Institutional Review Board and determined to be eligible for exempt review under 45 CFR 46.104(d)(1).

The composite examinations are computer-based examinations that cover material from several different courses, occur at two-week intervals, and are administered in a three-hour timeframe throughout the five semesters of the didactic curriculum. The composite examination process has been previously described in greater detail [14]. Table 1 includes the number of questions each course had on the examinations and the modality of proctoring (i.e., in-person vs. remote). Of note, examination 5 was not proctored and thus excluded from this study. 

### 2.3. Study Population and Data Collection

All P1 (*n* = 192) and P2 (*n* = 195) students enrolled in spring 2020 at a single 4-year, public PharmD program were included and served as their own controls in the before-after analysis. Students were excluded from the analysis if they did not take any composite examinations with remote proctoring.

The transition time point for this study was defined as when remote proctoring began. The first four examinations used in-person proctoring methods, while the last three examinations of the semester were proctored via the remote process described above.

Student scores on composite examinations administered before and during the remote learning period were collected retrospectively. Student age, gender, race, pharmacy school cumulative GPA prior to the spring 2020 semester, and Cognitive Test Anxiety Scale—Second Edition (CTAS-2) scores that had been obtained previously were also obtained retrospectively.

The CTAS-2 instrument includes 24 items to measure trait testing anxiety [15]. CTAS-2 scores were used to objectively compare examination performance amongst students with varying levels of test-taking anxiety. One study on student pharmacists and CTAS-2 scores has been published to date and demonstrated that the scale has a high internal consistency (Cronbach’s alpha = 0.9) and validity [16]. All students complete the survey upon matriculation into the PharmD program. For this study, students were classified into one of three groups based upon CTAS-2 severity. The cut-off points of low (24–43), moderate (44–66), and high (67–96) have been previously published and validated [17].

### 2.4. Statistical Analysis

All statistical analyses were performed using IBM SPSS Statistics for Windows, Version 25.0 (IBM Corporation, Armonk, NY, USA). Descriptive statistics were calculated for all variables. Shapiro–Wilk tests and scatterplots were examined to assess normality of the paired data in each cohort. Examination scores were all determined to be non-normally distributed for both the P1 and P2 cohorts, and thus, the appropriate non-parametric test was applied. Specifically, Wilcoxon signed rank tests compared median examination scores before and after the transition point. Kruskal-Wallis tests compared the change in composite examination scores amongst the CTAS-2 severity groups. Mann-Whitney tests compared the change in composite examination scores before and after the transition for the upper versus lower 50% of each cohort, as determined by cumulative GPA prior to the spring 2020 semester. All tests were two-tailed with an *a priori* level of significance of *p* < 0.05.

## 3. Results

A total of 190 P1 students and 194 P2 students were included in the analysis. Three students were excluded due to mid-semester attrition from the PharmD program (*n* = 2 in P1 cohort; *n* = 1 in P2 cohort) and thus had no participation in the remotely proctored examinations.

Demographic characteristics of participants are shown in Table 2. Most participants were female and White with a mean age of approximately 25 years.

**Figure 1:**
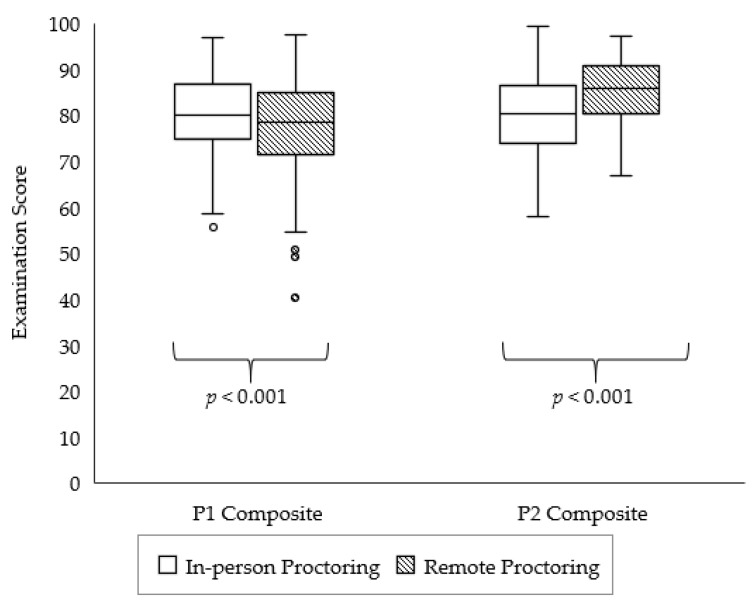
Comparison of spring 2020 composite examination scores for first-year (P1) and second-year (P2) student pharmacists with in-person versus remote proctoring.

Figure 1 shows composite examination scores with in-person versus remote proctoring. After transitioning to remote proctoring, composite examination scores significantly decreased for P1 students (*p* < 0.001). Conversely, composite examination scores significantly increased for P2 students after transitioning to remote proctoring (*p* < 0.001).

**Figure 2:**
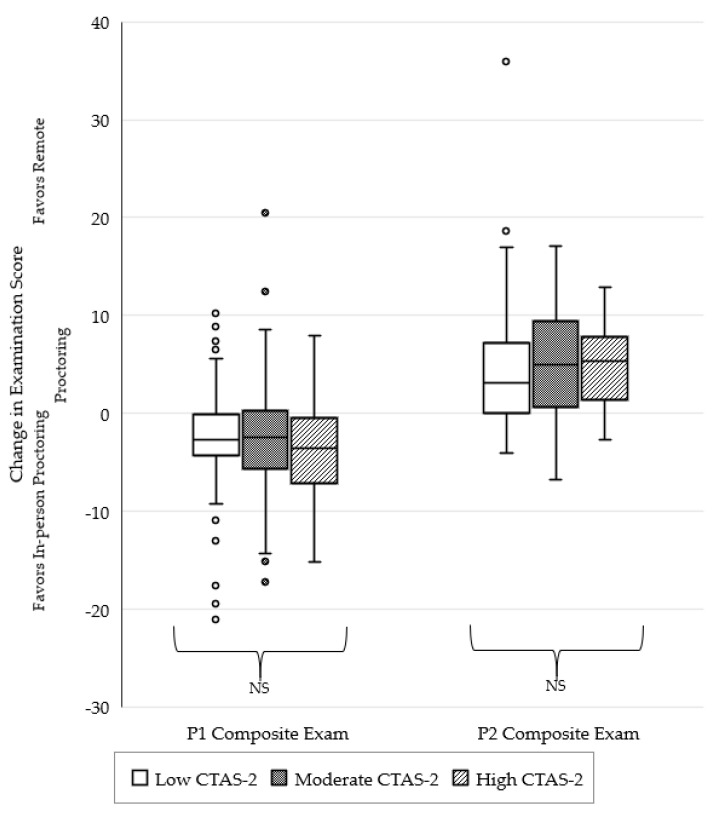
Change in examination performance after the transition to remote proctoring stratified by severity of student testing anxiety as determined by CTAS-2 score.

The change in examination performance after the transition to remote proctoring, with students stratified by CTAS-2 score, is depicted in Figure 2. There were no significant differences observed between the three CTAS-2 severity groups for P1 student pharmacists (*p* = 0.508). There were also no significant differences observed between the three CTAS-2 severity groups for P2 student pharmacists (*p* = 0.238).

**Figure 3:**
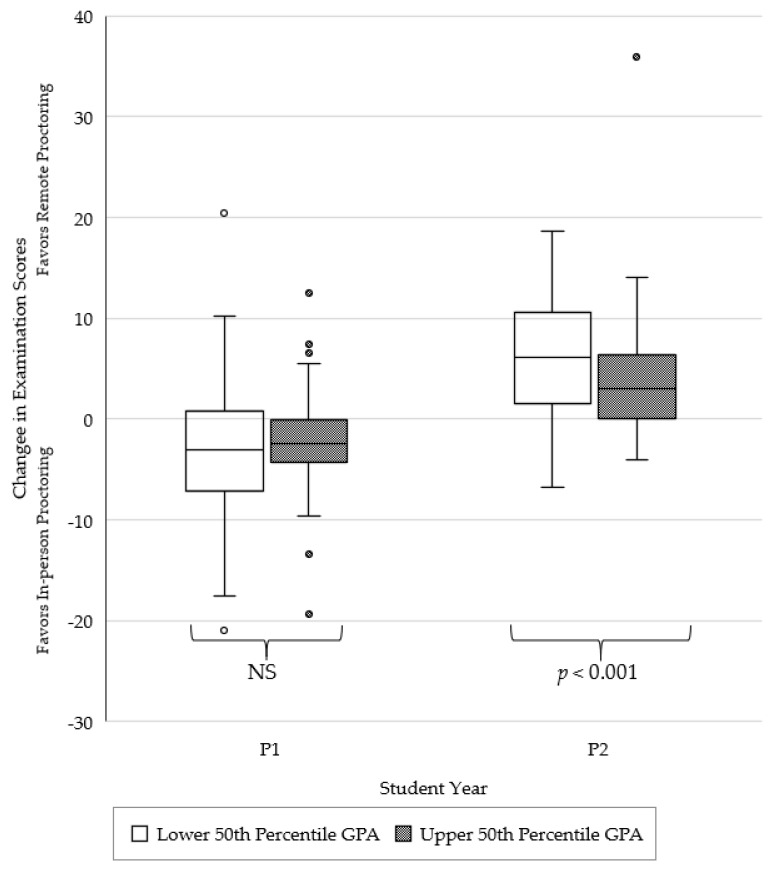
Change in examination performance after the transition to remote proctoring, stratified by student GPA.

Change in examination performance after the transition to remote proctoring, with student pharmacists stratified by GPA, is depicted in Figure 3. For P1 students, there were no significant differences in changes in examination scores when comparing the lower versus the upper 50th percentile GPA group (*p* = 0.248). However, significant differences in examination scores after the transition to remote proctoring were observed when comparing the lower versus the upper 50th percentile GPA group for P2 students (*p* < 0.001). Specifically, the lower 50th percentile GPA group had a significant increase in composite examination score as compared to their upper 50th percentile GPA peers.

## 4. Discussion

This study evaluated changes in academic performance among P1 and P2 students in the didactic PharmD curriculum at a single institution. Changes in academic performance were assessed by comparing examination performance prior to and after a transition to remote learning and proctoring due to the COVID-19 pandemic. The study results indicate that the impact of remote proctoring on examination scores may vary depending upon the student’s year within the curriculum as well as the student’s cumulative GPA. In this study, remote proctoring was not observed to significantly impact academic performance when comparing students with varying levels of testing anxiety.

Our study contributes to the growing evidence regarding how remote proctoring during the COVID-19 pandemic may impact student academic performance. A study of undergraduate students enrolled in a remote psychopharmacology course during the pandemic in Brazil found that students with higher psychological distress scores were less likely to change from an incorrect to a correct answer [18]. In our study, academic performance was observed to decrease for P1 students but increase for P2 students with the transition to remote proctoring. This may be secondary to P2 students being more familiar with remote learning technology than P1 students. P1 students are all located at a single campus, but P2 students are at one of three campuses spread across the state. Distance technology is necessary to connect these campuses and has been used throughout the curriculum even prior to the pandemic. This hypothesis is supported by a publication by Peimani and colleagues where technology was noted to be a major aspect influencing student perceptions of learning experiences during the pandemic [19]. Technology will likely remain a major factor influencing student perceptions of remote learning experiences as society progresses into the post-COVID-19 era.

It has been previously reported that students with higher testing anxiety tend to perform worse on examinations that were proctored remotely as compared to those proctored and administered in person [20]. In our study, academic performance did not differ based on a student’s level of testing anxiety (i.e., CTAS-2 score) for both P1 and P2 students. This study is the first to our knowledge showing that examination performance in student pharmacists does not change significantly with remote proctoring when considering the student’s level of testing anxiety. Each student experiences anxiety differently, as was demonstrated in a study of remote examination delivery for medical students during the pandemic [21]. Although this study did not show a significant change in academic performance based on testing anxiety, a student’s level of testing anxiety should be considered and assessed when altering examination processes.

When students were stratified by GPA in our study, no significant changes in academic performance after transitioning to remote proctoring were seen for P1 students; however, a significant improvement in academic performance was noted for P2 students with a GPA in the lower 50th percentile. Academic dishonesty is one possible explanation for the change in scores observed for P2 students. However, only a single instance of academic dishonesty was reported by a remote proctor during the remote proctoring period. A rigorous post-examination analysis was also conducted as part of the academic dishonesty detection process. This process included a hierarchical clustering analysis to detect similarity of student examination responses and an analysis of various data available from ExamSoft, including timestamped examination-taker activity and IP addresses for submissions and uploads [7].

Our observations regarding impact on academic integrity are contrary to those of another study where ad hoc online testing implemented during the pandemic was found to have negative implications on academic integrity [6]. A different study found that 52% of students perceived there to be no difference in ease of cheating for online vs. traditional assessments [22]. Thus, the currently available evidence is inconclusive regarding whether academic dishonesty is more, less, or equally problematic with online examinations; our study adds to this body of literature with only one reported instance of academic dishonesty in the remote examination setting.

An alternative possible reason for the improvements in academic performance during the remote proctoring period that were observed for P2 students with a GPA in the lower 50th percentile is that P2 students may be more comfortable with the rigors of the program’s curriculum and the composite examination structure as compared to P1 students. Having more experience with these aspects of the PharmD program may have helped P2 students adapt with greater ease to the remote testing procedure and thereby perform better academically. In addition, P2 students may also be more accustomed to the technology used for distance education given their longer time of enrollment within the program. Our campus consists of three campuses split across two adjacent time zones, and remote learning technology, including Zoom, is used regularly to facilitate learning, even prior to the pandemic. Technology and a student’s familiarity with it has been reported to be a major aspect influencing student learning during the pandemic [19].

Student preference for in-person versus remote proctoring is another factor to consider when interpreting our study results. A survey of students in medicine, dentistry, pharmacy, nursing, and applied medical sciences during the COVID-19 pandemic found that approximately one-third of students preferred remote examinations over in-person, and preference for remote examinations was significantly associated with GPA [23]. Our study mirrors these findings with significant changes in scores for P2 students with a GPA in the lower 50th percentile demonstrated.

Evidence of stress and anxiety due to the pandemic itself has been previously demonstrated in both the general population and student pharmacists [24,25,26] and should be considered within the context of our results. A study of undergraduate students (*n* = 2031) reported that less than half of participants were able to cope adequately with stress related to the COVID-19 pandemic [27]. However, another study, also conducted during the COVID-19 pandemic, demonstrated that health professions students have higher rates of resilience compared with their peers not pursuing a health profession [28]. In our study, student CTAS-2 scores were assessed pre-pandemic; it is important to consider that those scores could have changed during the pandemic due to stress from the pandemic itself.

There are limitations to this study to consider. One such limitation lies within the data analyzed for the P2 students. The spring semester for P2 students includes two modular courses that integrate pharmacotherapeutics, medicinal chemistry, and pharmacology: infectious disease (ID) and surgery, critical care, and transplant (SCCT). ID occurs in the first half of the semester and is assessed on examinations 1–5, while SCCT is in the latter half of the semester and assessed on examinations 6 through 8. Students may have found one modular course to be more challenging than the other, which may limit the ability to compare scores before and after the transition to remote proctoring around examination 5. However, the historical performance in these two courses has been comparable to performance included in this analysis. A second limitation is the study’s generalizability given that only a single PharmD program was included. The study sample mirrors the overall population of student pharmacists in the U.S., which is 63% female and 16.2% minority, supporting that this data is generalizable.

Lastly, confounding factors may exist. One such factor to consider is the simultaneous change to remote instruction in spring 2020. However, course and examination content as well as examination construction remained the same with the transition to remote instruction. The COVID-19 pandemic has led to a heightened awareness of the “digital divide” in many areas, including education [29,30,31]. Thus, another confounding factor to consider is the potential for inequality in digital accessibility among student pharmacists in this study. To help alleviate this potential inequality, students were offered the option to test on campus in a socially distant manner, and video-capable devices were available for students to use if needed.

Additional studies should be conducted to further investigate the potential impacts of remote proctoring modalities and a student’s level of testing anxiety. Data from other PharmD programs are needed to evaluate remote proctoring effects on pharmacy student performance. Beyond the didactic curriculum, further research may consider examining experiential education performance. This may include exploring differences in learning and academic performance for student pharmacists who completed the didactic curriculum with assessments delivered in a remote manner as compared to those students who did not complete their didactic curriculum during the global pandemic.

## 5. Conclusions

This study indicates that examination scores do not consistently improve or decline in student pharmacists after introducing remote proctoring. Furthermore, these findings suggest that remote proctoring procedures may impact students differently depending upon their year within the program. Remote proctoring was not observed to impact academic performance amongst students with varying levels of testing anxiety; however, a significant increase in score with remote proctoring was noted for P2 students within the lower 50th percentile GPA group, but no change was noted for P1s when stratified by GPA. Additional studies are needed to further characterize the impact of remote proctoring practices on academic performance in pharmacy education.

## Figures and Tables

**Table 1:** Composite examination structure and proctoring modality.

		Composite Examination with In-Person Proctoring				Composite Examination with Remote Proctoring		
Year	Course	1	2	3	4	6	7	8
No. of Questions								
P1	Cardiology	40	52	36	44	42	32	35
	D4	16	12	16	16	12	12	0
	PK	20	20	19	23	20	24	20
	*Total Questions*	*76*	*84*	*71*	*83*	*74*	*68*	*55*
P2	LESD	8	16	16	16	16	16	0
	ID	64	48	56	52	0	0	0
	SCCT	0	0	0	0	44	52	48
	*Total Questions*	*72*	*64*	*72*	*68*	*60*	*68*	*48*

D4, drug dosage, design, and delivery; ID, infectious diseases; LESD, literature evaluation and study design; P1, first-year student pharmacist; P2, second-year student pharmacist; PK, pharmacokinetics; SCCT, surgery, critical care, and transplant.

**Table 2:** Participant demographic characteristics.

	P1 Students (*n* = 190)	P2 Students (*n* = 194)
Age, mean (SD)	24.6 (4.09)	24.8 (2.93)
Gender, n (%)		
Female	125 (65.8)	116 (59.8)
Male	65 (34.2)	78 (40.2)
Race, n (%)		
White	119 (62.6)	136 (70.1)
Minority	69 (36.3)	53 (27.3)
Not reported	2 (1.1)	5 (2.6)
GPA, mean (SD)	3.25 (0.48)	3.15 (0.52)
CTAS-2, mean (SD)	52.6 (15.9)	50.4 (16.5)

CTAS-2, Cognitive Test Anxiety Scale—Second Edition; GPA, grade point average; P1, first-year student pharmacist; P2, second-year student pharmacist.

## Data Availability

Data from this study are not available for sharing due to ethical, legal, and privacy restrictions.
